# Polycomb group protein expression during differentiation of human embryonic stem cells into pancreatic lineage *in vitro*

**DOI:** 10.1186/1471-2121-15-18

**Published:** 2014-05-24

**Authors:** Prasad Pethe, Punam Nagvenkar, Deepa Bhartiya

**Affiliations:** 1 Stem Cell Biology Department, National Institute for Research in Reproductive Health, J.M. Street, Parel-12, Mumbai, India

**Keywords:** hES cells, PcG proteins, Differentiation, Definitive endoderm, Pancreatic progenitors

## Abstract

**Background:**

Polycomb Group (PcG) proteins are chromatin modifiers involved in early embryonic development as well as in proliferation of adult stem cells and cancer cells. PcG proteins form large repressive complexes termed Polycomb Repressive Complexes (PRCs) of which PRC1 and PRC2 are well studied. Differentiation of human Embryonic Stem (hES) cells into insulin producing cells has been achieved to limited extent, but several aspects of differentiation remain unexplored. The PcG protein dynamics in human embryonic stem (hES) cells during differentiation into pancreatic lineage has not yet been reported. In the present study, the expression of *RING1A, RING1B, BMI1, CBX2, SUZ12, EZH2, EED* and *JARID2* during differentiation of hES cells towards pancreatic lineage was examined.

**Results:**

*In-house* derived hES cell line KIND1 was used to study expression of PcG protein upon spontaneous and directed differentiation towards pancreatic lineage. qRT-PCR analysis showed expression of gene transcripts for various lineages in spontaneously differentiated KIND1 cells, but no differentiation into pancreatic lineage was observed. Directed differentiation induced KIND1 cells grown under feeder-free conditions to transition from definitive endoderm (Day 4), primitive gut tube stage (Day 8) and pancreatic progenitors (Day 12-Day 16) as evident from expression of SOX17, PDX1 and SOX9 by qRT-PCR and Western blotting. In spontaneously differentiating KIND1 cells, *RING1A* and *SUZ12* were upregulated at day 15, while other PcG transcripts were downregulated. qRT-PCR analysis showed transcripts of *RING1B, BMI1*, *SUZ12*, *EZH2* and *EED* were upregulated, while *RING1A* and *CBX2* expression remained low and JARID2 was downregulated during directed differentiation of KIND1 cells. Upregulation of BMI1, EZH2 and SUZ12 during differentiation into pancreatic lineage was also confirmed by Western blotting. Histone modifications such as H3K27 trimethylation and monoubiquitinylation of H2AK119 increased during differentiation into pancreatic lineage as seen by Western blotting.

**Conclusion:**

Our study shows expression of PcG proteins was distinct during spontaneous and directed differentiation. Differentiation into pancreatic lineage was achieved by directed differentiation approach and was associated with increased expression of PcG proteins RING1B, BMI1, EZH2 and SUZ12 accompanied by increase in monoubiquitinylation of H2AK119 and trimethylation of H3K27.

## Background

Differentiation of hES cells requires precise orchestration of modifications at chromatin levels, mitochondrial level and changes in cell architecture. Chromatin remodeling is an essential step towards differentiation in the case of pluripotent hES cells [1,2]. Embryonic stem cells have open chromatin structure which enables them to differentiate into any lineage [3,4]. Two most widely studied chromatin modifications in ES cells are (i) DNA methylation catalyzed by enzymes like DNMT3a, DNMT3b, DNMT1 [5] and (ii) histone modifications [6,7] carried out by Polycomb group (PcG) proteins and Trithorax group proteins (TxG). The histone modifications by PcG proteins prevent precocious differentiation of ES cells [8]. PcG proteins may play an important role in the early development, while DNA methylation is involved in gene silencing in differentiated cells [1].

PcG proteins modify histones by methylation (mono, di, tri methylation) and ubiquitinylation, to bring about repression of genes [9-12]. PcG form multiprotein complexes called Polycomb Repressive Complexes (PRC) such as PRC1 and PRC2 [8,13,14]. The PRC1 complex comprises of CBX, BMI1, RING1A, RING1B, PHC, which catalyze monoubiquitinylation of lysine 119 on histone H2A(H2AK119ub1) [15,16]. Loss of PRC1 protein RING1B is embryo lethal, knockout animals for other PRC1 protein are not embryonically lethal but have several developmental defects [17-19]. The core PRC2 comprises of SUZ12, EED and EZH2 which catalyze the addition of di-methyl or tri-methyl groups to lysine 27 on histone H3 (H3K27me3). PRC2 is essential for embryonic development as evident from knockout mice studies, which show that loss of PRC2 proteins like SUZ12, EZH2 and EED results in embryonic lethality [20-23].

Apart from their requirement for overall development, PcG proteins are involved in proliferation and development of specific cell types like skeletal muscles, neural stem cells, hematopoietic cells [20,24-26]. PcG proteins such as BMI1 and EZH2 are often found to be overexpressed in cancer cells [27-29].

Hence, it becomes important to study the dynamics of polycomb group proteins during development of various cell types in humans. The best model to study the PcG expression during early development in humans is differentiating embryonic stem cells. hES cells are pluripotent cells with potential to differentiate into all three lineages viz. endoderm, mesoderm and ectoderm [30,31]. Previous reports have studied PcG proteins in ES cells differentiating into ectoderm and mesoderm [9,32,33]. Recently polycomb mediated histone modifications (H3K27me3 and H3K4me3) at key genes during differentiation of hES cells into pancreatic lineage was reported [34]. One of the important area of research has been differentiation of hES cells into functional islet cells for treatment of type I diabetes [26,35-37]. Even though PcG proteins are crucial during differentiation, there are no reports available as yet of dynamics of PcG expression during differentiation into pancreatic lineage.

In the present study, *in-house* derived hES cell line KIND1 [30,38] was differentiated into pancreatic lineage under feeder-free culture system by two strategies viz spontaneous and directed differentiation. The expression of PcG protein transcript *RING1A, RING1B, BMI1, CBX2, SUZ12, EZH2, EED* and *JARID2* was examined during differentiation of KIND1 and compared to the pattern in adult human pancreatic RNA.

## Results

### Differentiation of KIND1 hES cells

#### Spontaneous differentiation

**Figure 1:**
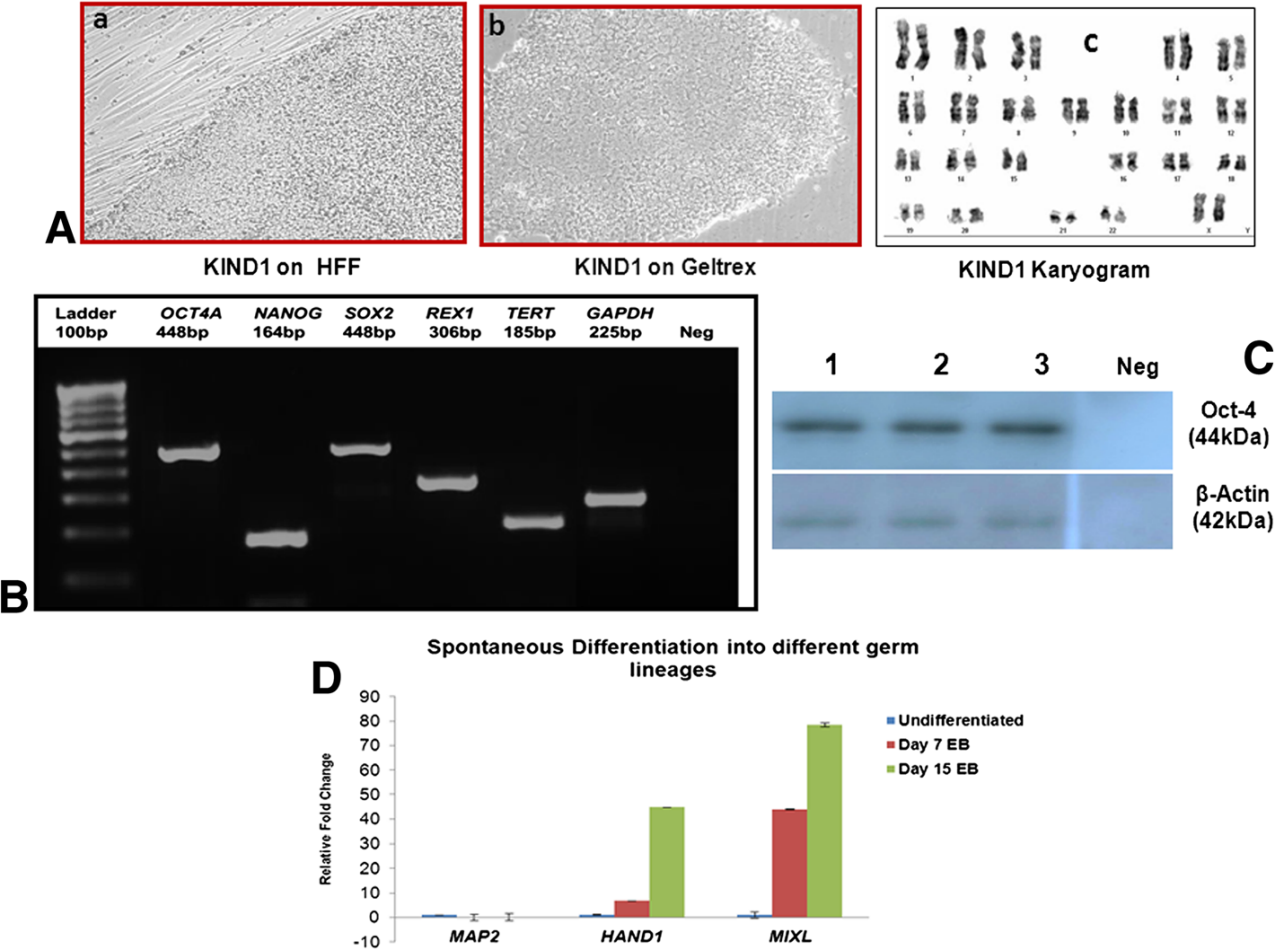
**Adaptation of KIND1 cells to feeder free culture system & characterization of feeder free KIND1 cells & embryoid body (EB) differentiation. (A)** Bright field images of KIND1 cells cultured on HFF (a) and geltrex (b) Magnification 10X. (c) Cytogenetic analysis of KIND1 exhibiting normal 46, XX chromosome complement. **(B)** RT-PCR analysis of pluripotency specific gene transcripts **(C)** Western Blot analysis for OCT4 in undifferentiated feeder free KIND1 cell lysate loaded in triplicate with β actin as loading control. **(D)** Expression of representative gene transcripts of ectoderm (*MAP2*), mesoderm (*HAND1*) and endoderm (*MIXL*) in Day 7 and Day 15 EBs grown in suspension culture. The expression is with respect to undifferentiated KIND1 cells (set as 1). Error bars represent ± Standard Error of Mean (SEM).

For spontaneous differentiation, embryoid bodies (EBs) were generated from KIND1 cells as described earlier [38]. Three representative genes *MAP2* (ectoderm lineage), *HAND1* (mesoderm lineage) and *MIXL* (endoderm lineage) were studied by qRT-PCR from embryoid bodies harvested on day 7 and day 15. Expression of *MIXL* and *HAND1* gene transcripts was seen, but the expression of *MAP2* was low (Figure 1D). However, the embryoid bodies did not show expression of gene transcripts specific to pancreatic lineage such as *PDX1*, *SOX9*, and *NKX6.1* (data not shown). Spontaneous differentiation did not yield cells of pancreatic lineage.

#### Directed differentiation

##### Feeder-free culture of KIND1 cells

**Figure 2:**
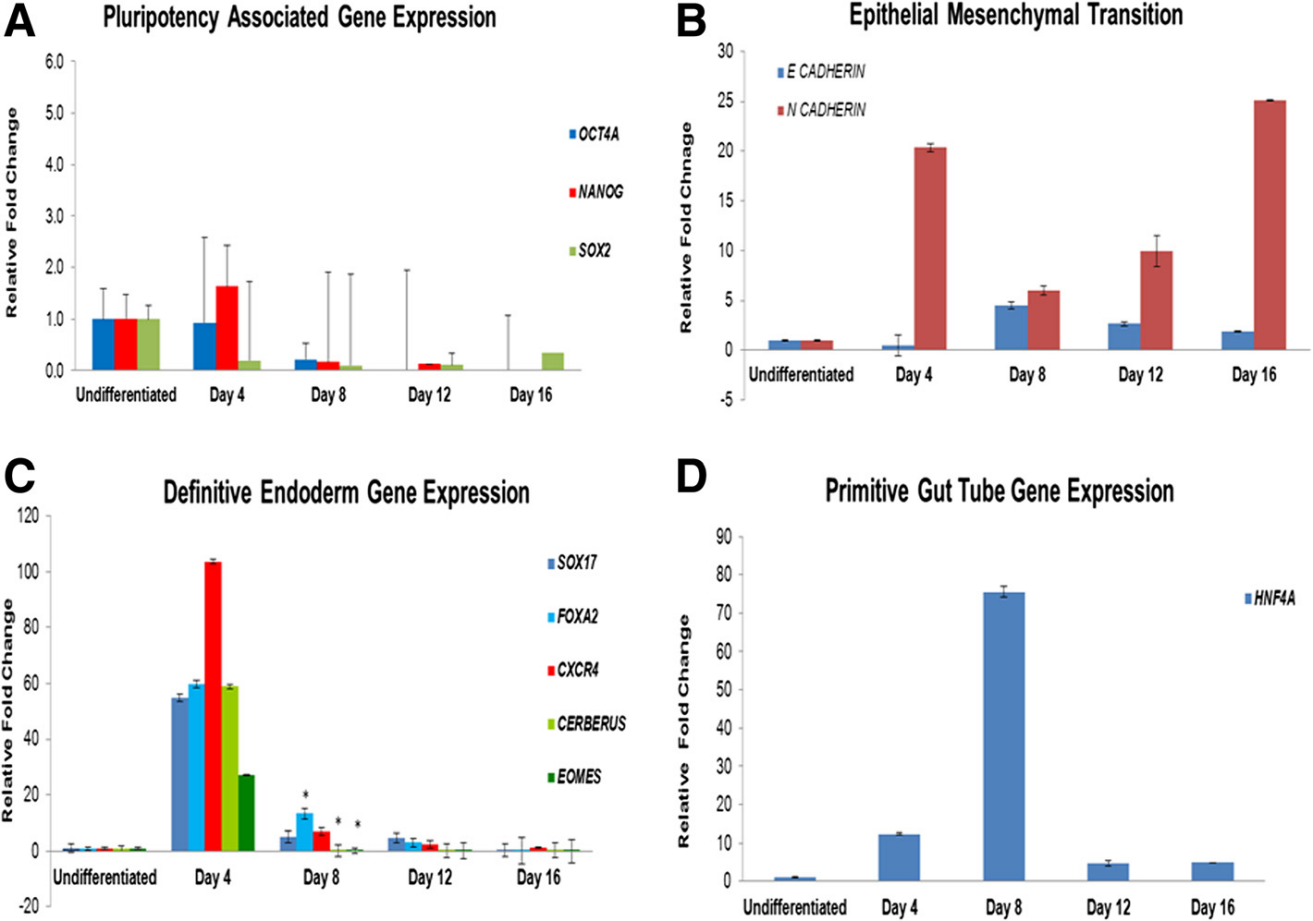
**Characterization of KIND1 cells differentiated into pancreatic lineage. (A)** Expression of pluripotency associated gene transcripts (*OCT4, NANOG, SOX2*) during differentiation of KIND1 cells from Day 4 – Day 16. **(B)** Expression of *E CADHERIN* and *N CADHERIN* during differentiation in undifferentiated and Day 4 – Day 16. **(C)** Expression of definitive endoderm specific gene transcripts (*SOX17, FOXA2, CXCR4, CERBERUS, EOMESODERMIN*) in undifferentiated and Day 4 – Day 16. **(D)** Expression of primitive gut tube marker *HNF4A* in undifferentiated and Day 4 – Day 16. The expression of all gene transcripts is relative to undifferentiated KIND1 cells (set as 1). Error bars represent ± SEM, statistical significance represented as *(p <0.05).

To avoid interference from factors secreted by feeder cells during directed differentiation, KIND1 cells were cultured on reduced basement matrix Geltrex. KIND1 cells growing on human feeder fibroblast (HFF) [30] were shifted to grow on Geltrex coated culture surface (Figure 2A {b}). The feeder-free KIND1 colonies are circular and large compared to colonies on HFF. Feeder-free KIND1 cells were regularly characterized for pluripotency markers at both protein and transcript levels at various passages. Feeder-free KIND1 cells showed expression of *OCT4A*, *NANOG*, *SOX2*, *REX1* and *TERT* by RT-PCR (Figure 1B) and OCT4A protein expression was seen by Western blot (Figure 1C). These cells also showed a normal karyotype post feeder-free culture (Figure 1A {c}). Thus the KIND1 cells retain the pluripotency characteristics post feeder free culture.

##### Differentiation of feeder-free *KIND1 cells into pancreatic lineage*

**Figure 3:**
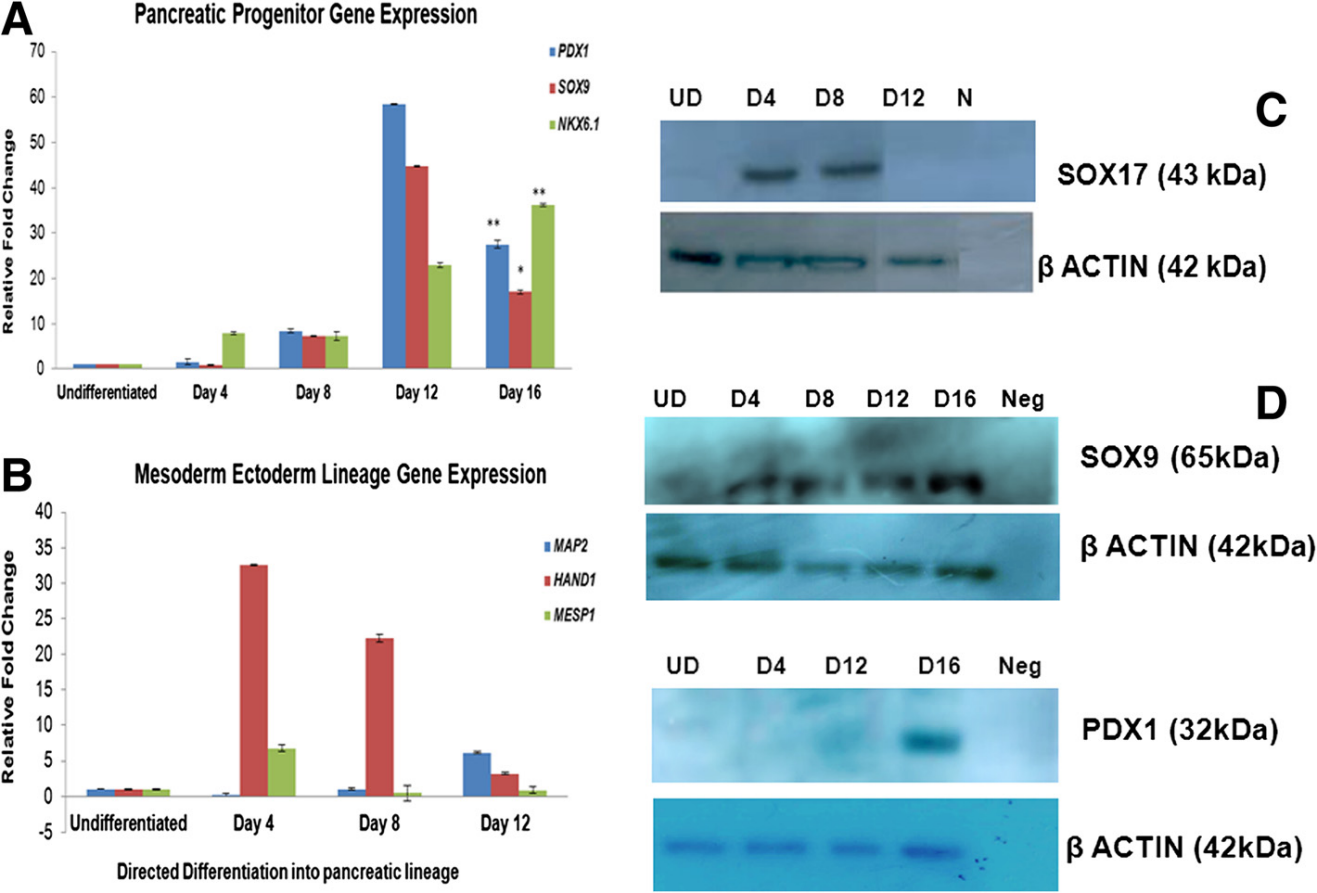
**Characterization of KIND1 cells differentiated into pancreatic lineage by directed differentiation approach*. *(A)** Expression of pancreas specific gene transcripts (*PDX1. SOX9, NKX6.1*) in undifferentiated and Day 4 – Day 16. **(B)** Expression of representative gene transcripts of ectoderm (*MAP2*) and mesoderm (*HAND1*, *MESP1)* in undifferentiated and Day 4 – Day 16. The expression is relative to undifferentiated KIND1 cells (set as 1). Error bars represent ± SEM, statistical significance represented as *(p < 0.05) and **(p < 0.02). **(C)** Western Blot analysis for SOX17 protein in cell lysates from undifferentiated (UD), Day 4 (D4), Day 8 (D8), and Day 12 (D12) samples, with β ACTIN as housekeeping protein. **(D)** Western Blot analysis for PDX1 and SOX9 protein in cell lysates from undifferentiated (UD), Definitive Endoderm Day 4 (D4), Primitive Gut Tube Day 8 (D8), and pancreatic progenitors Day 12 (D12) - Day 16 (D16) samples, with β ACTIN as housekeeping protein.

Initiation of differentiation resulted in upregulation of *NANOG* at Day 4 while *SOX 2* gene transcript declined (Figure 2A). KIND1 cells underwent epithelial to mesenchymal transition evident from expression of *E-CADHERIN* and *N-CADHERIN* transcripts (Figure 2B). At Day 4, *N-CADHERIN* showed significant upregulation while E-*CADHERIN* was downregulated; later N-*CADHERIN* expression remained high as compared to undifferentiated feeder free KIND1 cells. Activin A treatment led to maximal expression of definitive endoderm (DE) specific genes like *SOX17*, *FOXA2*, *CXCR4*, *CERBERUS* and *EOMESODERMIN* at Day 4 and as the differentiation continued their expression declined (Figure 2C). Western Blot results for SOX17 also confirmed definitive endoderm formation (Figure 3C). *HNF4A* expression peaked at day 8 suggesting exit from the DE (definitive endoderm) stage and entry into primitive gut tube stage (Figure 2D). Peak expression of pancreas specific transcripts *PDX1*, *NKX6.1* and *SOX9* was seen between days 12–16 indicating presence of pancreatic lineage cells (Figure 3A). PDX1 and SOX9 protein expression was seen at Day 16 by Western blotting (Figure 3D). Expression of *MAP2* (ectoderm specific), *HAND1* and *MESP1* (mesoderm specific) transcripts during directed differentiation was found to be low at day 12 (Figure 3B) indicating cells differentiated primarily into endoderm lineage. Thus, directed differentiation resulted in the formation of cells of pancreatic lineage.

#### Polycomb group protein dynamics during differentiation

We first examined the expression of PcG expression in KIND1 cultured on HFF and under feeder free system, followed by studying PcG expression in differentiated KIND1 cells.

##### KIND1 cultured on human feeder fibroblasts

**Figure 4:**
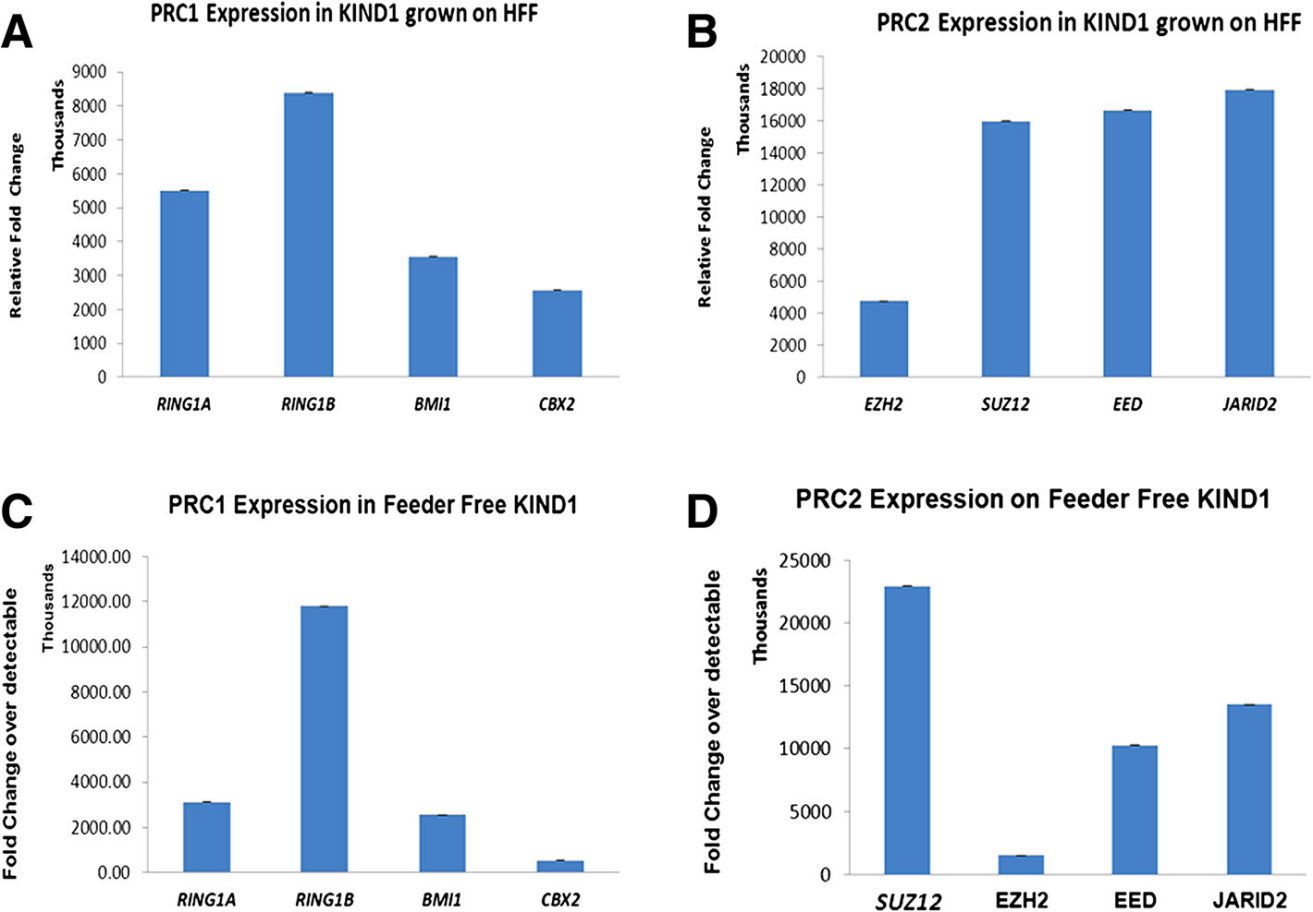
**Expression of PcG protein transcripts in KIND1 cells grown on HFF and feeder free KIND1 cells.** qRT-PCR results show expression of **(A)** PRC1 group members- *RING1A, RING1B*, *BMI1 and CBX2***(B)** PRC2 group members *SUZ12, EZH2, EED* and *JARID2* in KIND1 cells cultured on human feeder fibroblasts (HFF). qRT-PCR results showing expression of **(C)** PRC1 group members- *RING1A, RING1B*, *BMI1and CBX2***(D)** PRC2 group members *SUZ12, EZH2, EED* and *JARID2* in feeder free KIND1 cells. The expression is with respect to highest detectable (Ct 40). Error bars represent ± SEM.

*RING1B* expression was highest compared to *RING1A, BMI1* and *CBX2* in the PRC1 group, while among PRC2 members *SUZ12, JARID2* and *EED* expression was most prominent (Figure 4A and B).

##### Feeder-free KIND1 cells

The expression of PRC1 gene transcripts showed similar expression pattern to that seen when KIND1 cells were cultured on HFF. *RING1B* was significantly expressed more compared to *BMI1*, *RING1A*, *CBX2* while among the PRC2 *SUZ12* was expressed significantly more than *EZH2, JARID2* and *EED* (Figure 4C and D).

##### Spontaneous differentiation

**Figure 5:**
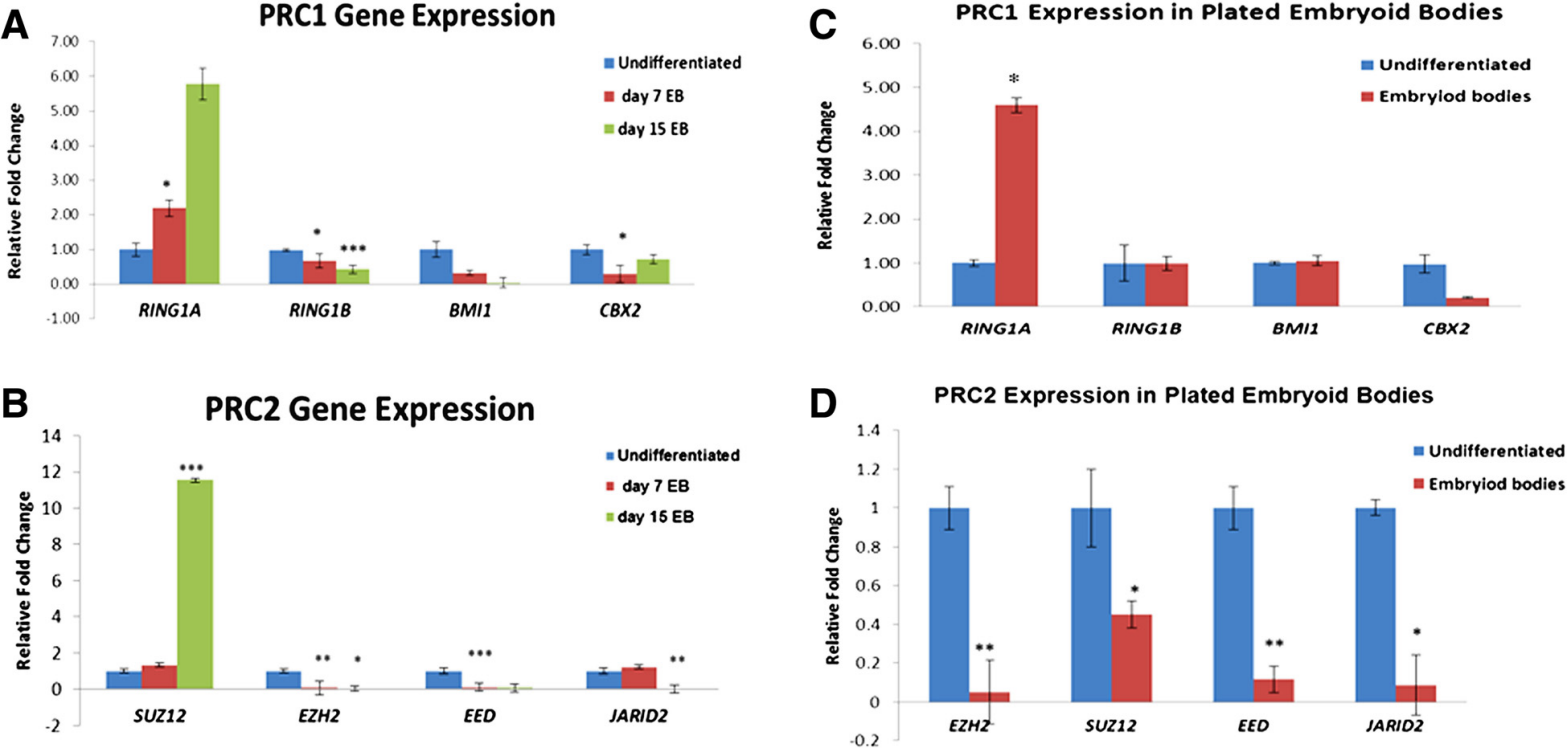
**Characterization of Embryoid Bodies (EBs) for PcG gene transcripts. (A)** Expression of PRC1 (*RING1A, RING1B*, *BMI1and CBX2*) in Day 7 and Day 15 EBs grown in suspension culture. **(B)** Expression of PRC2 (*EZH2, SUZ12, EED* and *JARID 2*) gene transcripts in Day 7 and Day 15 EBs grown in suspension culture. **(C)** Expression of PRC1 (*RING1A, RING1B*, *BMI1and CBX2*) gene transcripts at Day 10 in EBs cultured on gelatin coated dishes. **(D)** Expression of PRC2 (*EZH2, SUZ12, EED* and *JARID 2*) gene transcripts at Day 10 in EBs cultured on gelatin coated dishes. The expression is relative to undifferentiated KIND1 cells (set as 1). Error bars represent ± SEM, statistical significance represented as *(p < 0.05), **(p < 0.02) and ***(p < 0.001).

*RING1A* was expressed in high amounts during day 7 and day 15 of differentiation, while *RING1B, BMI1* and *CBX2* was significantly down regulated as compared to undifferentiated KIND1 cells (Figure 5A). Of the PRC2 members *SUZ12* gene transcripts was highly expressed at day 15, while *EZH2*, *EED* and *JARID2* were significantly down regulated compared to undifferentiated KIND1 cells (Figure 5B). To test whether the expression of PcG observed is due to suspension culture, the EBs after 7 days in suspension were plated for 10 days. Post 10 days, the expression of PRC2 gene transcripts *SUZ12*, *EZH2*, *EED* and *JARID2* were down regulated compared to undifferentiated cells (Figure 5C and D).

##### Directed differentiation

**Figure 6:**
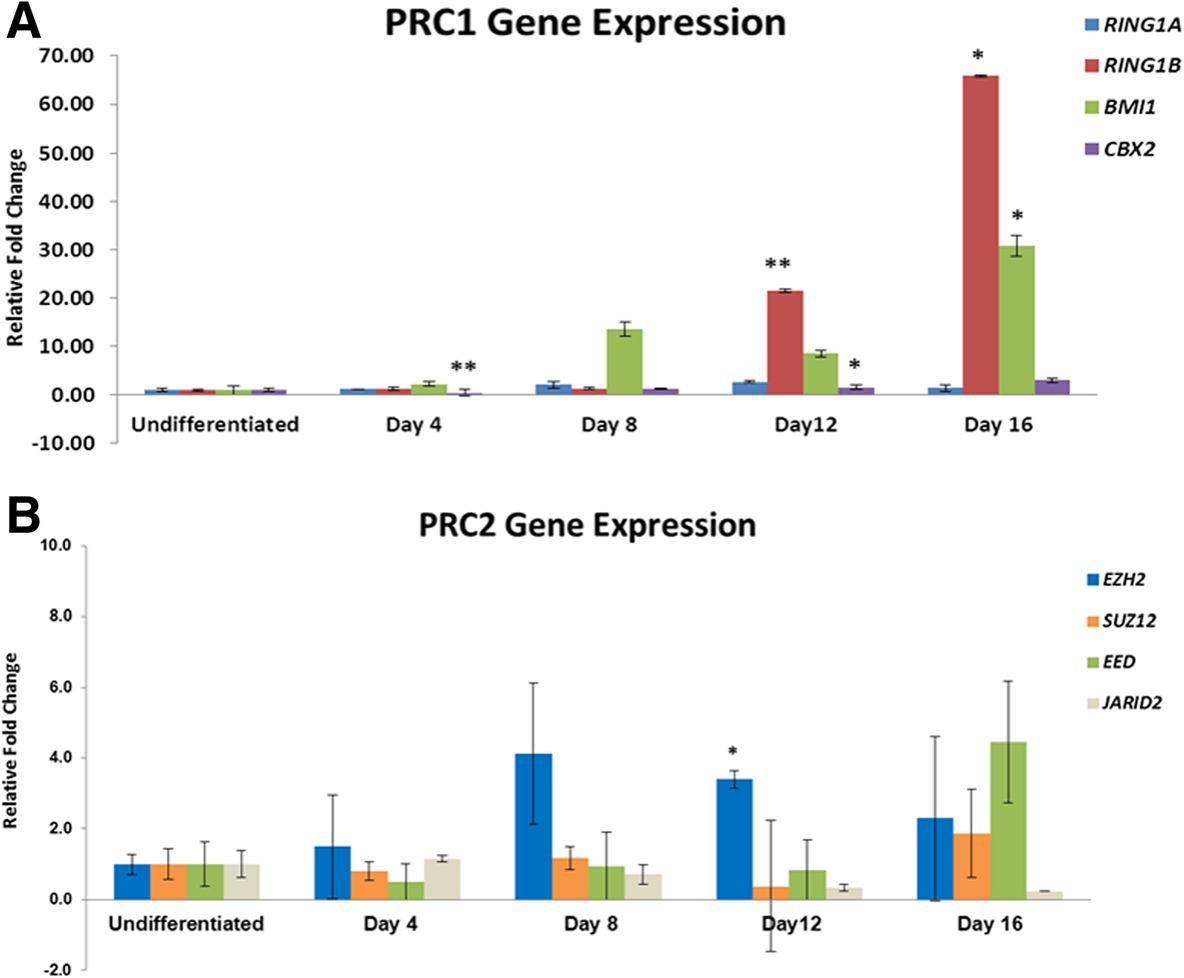
**Polycomb group protein expression during directed differentiation of KIND1 cells. (A)** PRC1 gene transcript (*RING1A, RING1B*, *BMI1and CBX2*) expression in undifferentiated and Day 4 – Day 16. **(B)** Expression of PRC2 gene transcripts (*EZH2, SUZ12, EED* and *JARID 2*) in undifferentiated and Day 4 – Day 16. The expression is relative to their expression in undifferentiated KIND1 cells (set as 1). Error bars represent ± SEM, statistical significance represented as *(p < 0.05), **(p < 0.02).

**Figure 7:**
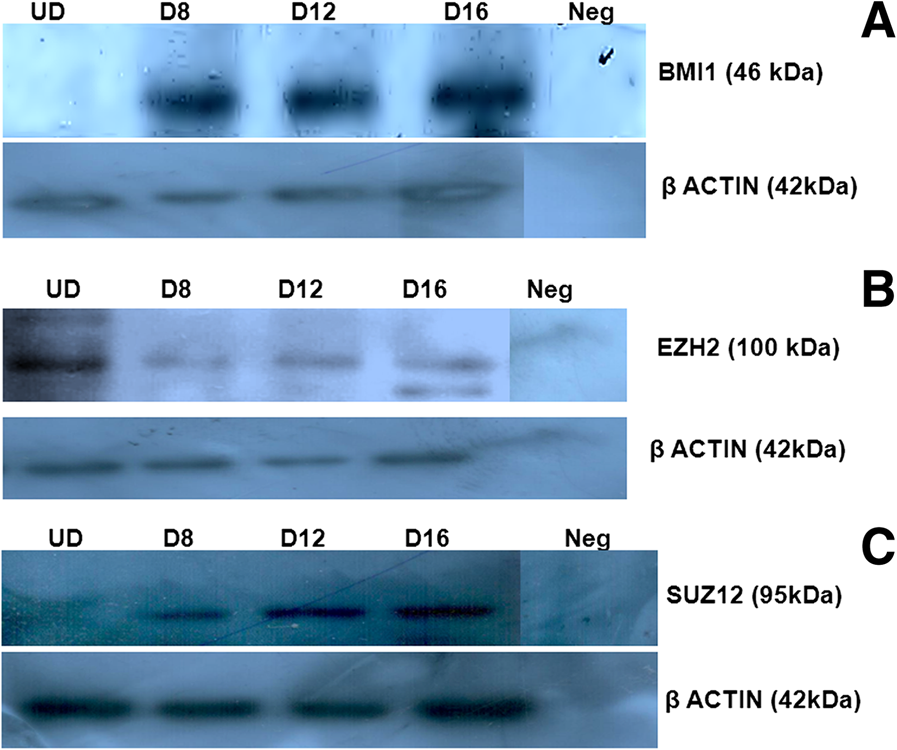
**Polycomb group protein expression during directed differentiation of KIND1 cells*.*** Western Blot analysis of **(A)** BMI1, **(B)** EZH2 and **(C)** SUZ12 proteins in undifferentiated KIND1 (UD), Day 8 (D8), Day 12 (D12) and Day 16 (D16) cell lysates during directed differentiation with β ACTIN as housekeeping protein.

With the onset of differentiation the expression of PRC1 and PRC2 proteins changed. PRC1 transcript *RING1B* and *BMI1* remained elevated over the entire duration, while *RING1A* and *CBX2* maintained low level of expression (Figure 6A). Western Blotting also showed that BMI1 expression increased steadily over the course of differentiation (Figure 7A). Among the PRC2 members, *SUZ12* gene transcript steadily increased as the cells differentiate into pancreatic lineage. Western Blotting for SUZ12 shows very low expression in undifferentiated KIND1 cells, but as the differentiation progresses SUZ12 expression increased (Figure 7C). *EZH2*, a key PRC2 member with methytransferase activity, at mRNA level peaked at day 8 (Figure 6B). At the protein level, EZH2 unlike SUZ12 was expressed in undifferentiated cells and its expression was seen till day 16 (Figure 7B). *EED* expression remained low as differentiation proceeded but was 4 fold high on Day 16 (Figure 6B). *JARID2* a member of Jumonji family, showed steady reduction in expression compared to undifferentiated KIND1 cells (Figure 6B).

##### Histone modifications

**Figure 8:**
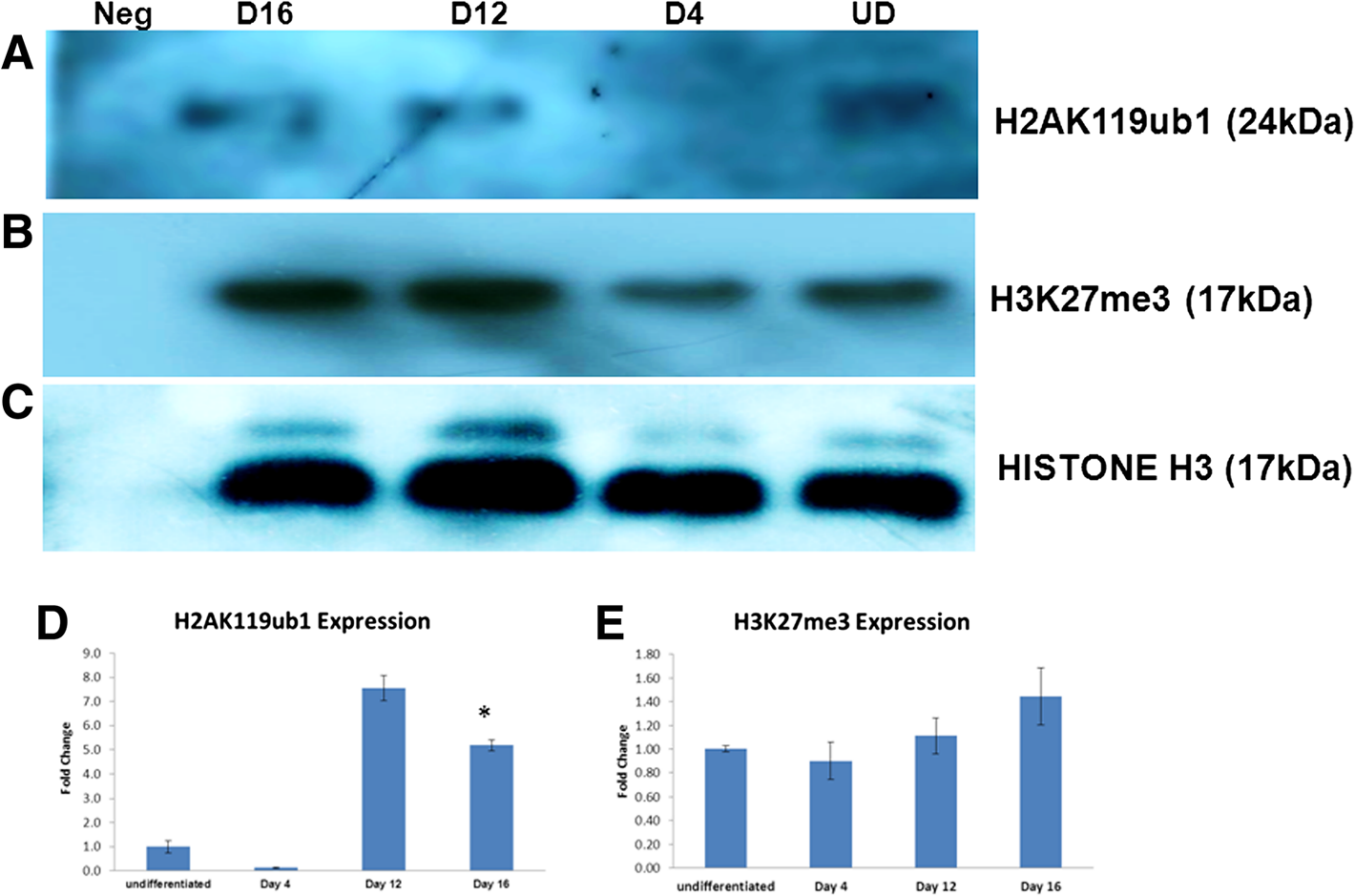
**Expression of H3K27me3 and H2AK119ub1 during directed differentiation of KIND1 cells.** Western Blot analysis of **(A)** H2AK119ub1 **(B)** H3K27me3 in undifferentiated KIND1 (UD) Day 4 (D4), Day 12 (D12) and Day 16 (D16) cell lysates during directed differentiation with **(C)** HISTONE H3 as housekeeping protein. Densitometric analysis of **(D)** H2AK119ub1 and **(E)** H3K27me3 expression during differentiation. Error bars represent ± Standard Error of Mean, statistical significance represented as *(p < 0.05).

Both PRC1 and PRC2 proteins repress genes by bringing about histone modifications. We observed increase in levels of Histone H2A monoubiquitinylation (Figure 8A). We also observed increase in trimethylation of H3K27 during directed differentiation (Figure 8B).

##### Expression of p19/ARF

**Figure 9:**
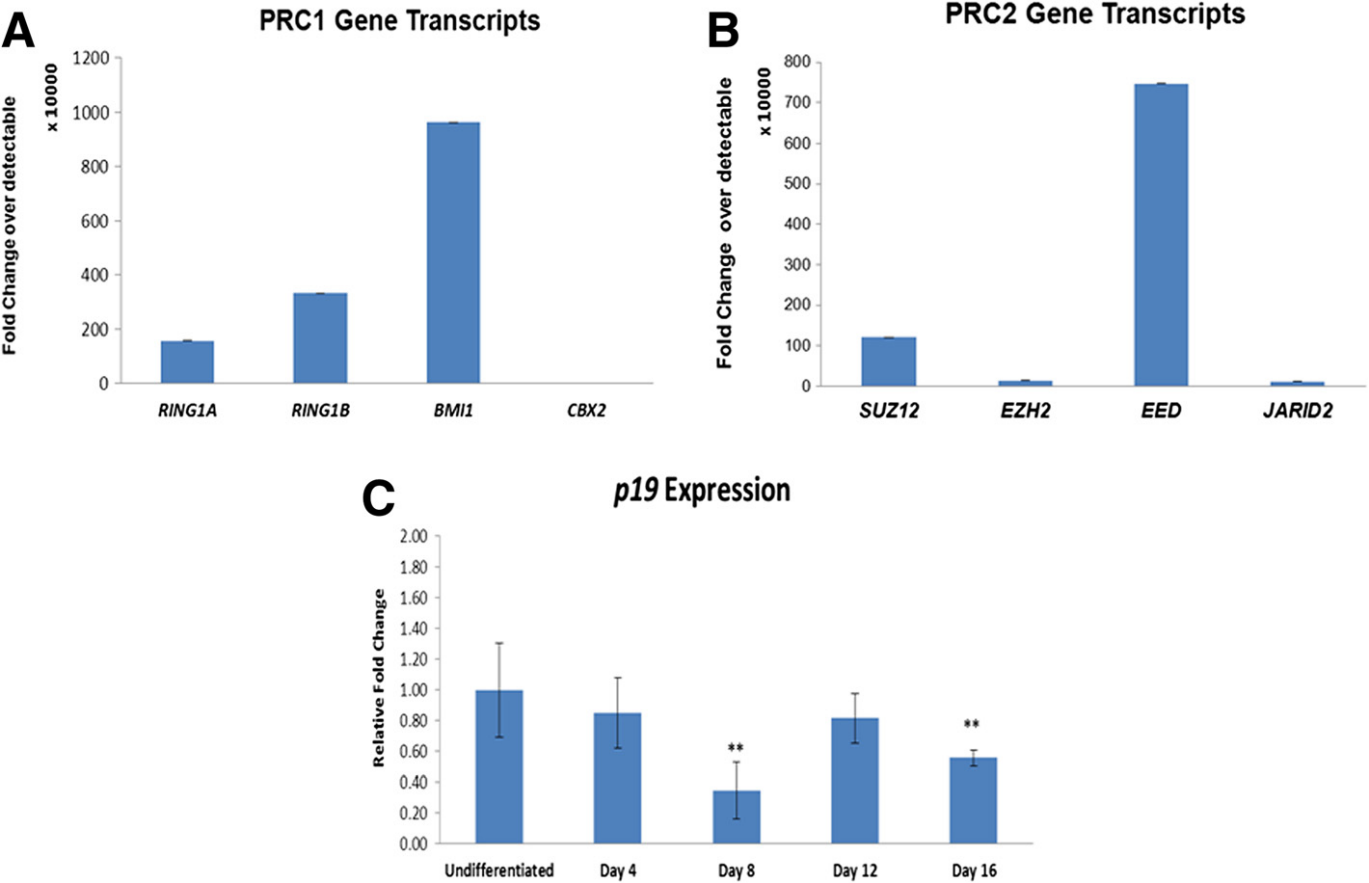
**Expression of PcG gene transcripts in adult human pancreas & p19 expression during differentiation into pancreatic lineage. (A)** Expression of PRC1 gene transcripts -*RING1A, RING1B*, *BMI1and CBX2 ***(B)** Expression of PRC2 gene transcripts -*EZH2, SUZ12, EED* and *JARID2* in adult human pancreas. The expression is with respect to highest detectable Ct 40. **(C)** Expression of *p19/ARF* in undifferentiated and Day 4 – Day 16, the expression is relative to undifferentiated KIND1 cells (set as 1). Error bars represent ± SEM, statistical significance represented as *(p < 0.05), **(p < 0.02).

The expression of p19 decreased significantly at day 8 and day 16 compared to undifferentiated KIND1 cells (Figure 9C). However, expression of p16/INK4a could not be detected in undifferentiated and differentiated KIND1 cells (data not shown).

#### Polycomb group protein expression in adult human pancreatic RNA

Of the PRC1 proteins, *BMI1* is expressed higher than *RING1A, RING1B and CBX2* (Figure 9A), while among PRC2 complex, *EED* and *SUZ12* is expressed to higher amounts while expression to *EZH2* and *JARID 2* transcripts is low (Figure 9B).

## Discussion

PRC2 group proteins have been shown to be dispensable for pluripotency; however they are required during differentiation shown by both *in vivo* and *in vitro* studies [32,39]. Though importance of PcG proteins during differentiation is known, their expression during differentiation of hES cells into pancreatic lineage has not yet been evaluated. To achieve this, we differentiated KIND1 via spontaneous differentiation and directed differentiation. EBs showed higher expression of endoderm and mesoderm gene transcript while lower expression of ectoderm gene transcripts was seen. However gene transcripts for *PDX1*, *SOX9* and *NKX6.1* were not detected in differentiated EBs. Hence, KIND1 cells were differentiated by directed differentiation which involves sequential addition of cytokines to generate cells of pancreatic lineage, and this necessitated culturing KIND1 cells under feeder free conditions. The PcG gene transcripts profile of feeder-free KIND1 cells was similar to KIND1 cells cultured on HFF cells.

Upregulation of pluripotency controlling gene *NANOG* on day 4 indicated that it may be required for endoderm differentiation, in contrast to its role in maintaining undifferentiated state. Previous reports have also shown that these transcription factors are involved in lineage specification and differentiation [40-42]. Gene transcripts of ectodermal marker (*MAP2*), cardiac mesoderm marker (*MESP1*) were seen at low level during pancreatic differentiation, indicating differentiation of KIND1 cells primarily into endoderm lineage. Pancreas specific gene transcripts *PDX1*, *SOX9* and *NKX6.1* were expressed during later stages of differentiation (day12-day16) which was also confirmed by Western blotting, indicating the differentiation of KIND1 cells into cells of pancreatic lineage by directed differentiation.

Once the differentiation of KIND1 into pancreatic lineage was achieved, we studied the expression of PcG proteins. *BMI1* has been shown to be important for self renewal and differentiation of neural stem cells and hematopoietic stem cells. BMI1 represses the expression of cell cycle inhibitors like p21, p19 and p16, leading to proliferation of stem cells [25,26,43,44]. *BMI1* was found to be highly expressed during directed differentiation of KIND1 cells into pancreatic lineage and this is being reported for the first time. We found the increased expression of BMI1 led to decrease in expression of p19/CDKN2D transcript. *CBX2* and *RING1A* gene transcript levels remain almost unaltered during differentiation, thus it suggests that CBX2 and RING1A may not play important role during endoderm differentiation. RING1B transcript was significantly upregulated during differentiation compared to undifferentiated KIND1 cells. We found the increased *RING1B* and *BMI1* led to increase in H2AK119ub1 levels during differentiation. Total adult human pancreatic RNA also showed higher expression of *BMI1* than *RING1A,* RING1B and CBX2 thus highlighting role of *BMI1* in differentiated endodermal cells.

In mice EZH2, SUZ12, EED and JARID2 knockout results in embryonic lethality [11,22,45,46]. Our data shows that *EZH2, EED* and *SUZ12* transcript expression increased compared to undifferentiated KIND1 cells during differentiation. On the other hand, *JARID 2* levels decreased steadily during both directed and spontaneous differentiation, similar to results obtained by earlier groups [7,34]. Studies carried out earlier have shown *EZH2, EED* decrease upon differentiation of mouse ES cells [6,47,48]. Also, previous reports that studied expression of PcG proteins in differentiated embryonic stem cells, have used spontaneous differentiation approach to induce differentiation [8,9,48,49]. Van Arensbergen et al. [50] reported that pancreatic progenitors have high levels of H3K27me3 which gives them plasticity to differentiate either to acinar or beta cell. PRC2 proteins EED, SUZ12 and EZH2 are actively involved in H3K27me3 modification [6,7]. Results of the present study show that *SUZ12, EZH2* and *EED* were upregulated during differentiation of KIND1 cells into pancreatic cells along with an increase in the H3K27me3 levels. The expression profile of PRC2 proteins at the transcript level observed in adult human pancreas is similar to that seen in KIND1 cells differentiated into pancreatic lineage.

The expression of *RING1B*, *BMI1, EZH2, EED*, and *SUZ12* gene transcripts during spontaneous and directed differentiated hES cells indicates that changes in PcG expression is not a generalized phenomenon observed during differentiation. The increase in expression of PcG proteins correlated with increase in trimethylation of histone H3K27 and monoubiquitinylation at H2AK119. Understanding the dynamics of polycomb group proteins during hES cell differentiation may help to design better differentiation strategies in future.

## Conclusions

We show that directed differentiation resulted in generation of cells of pancreatic lineage. Our data shows that expression of PRC1 and PRC2 members had distinct expression profile depending on whether KIND1 cells were subjected to directed or spontaneous differentiation. Expression of PRC1 transcripts *RING1B* and *BMI1*; PRC2 transcripts *SUZ12, EZH2* and *EED,* increased during pancreatic differentiation, which resulted in increase in H2AK119ub1 and H3K27me3 modification. PRC1 group protein RING1B and BMI1 and PRC2 group protein EZH2 and SUZ12 are expressed during differentiation into pancreatic lineage. This study will initiate more research on PcG proteins and their role in cell differentiation.

## Methods

### Human ES cells culture on HFF

All chemicals for cell culture were obtained from Invitrogen (Carlsbad, CA, USA), unless otherwise indicated. The culture and passaging was done as described earlier [30]. KIND1 colonies were passaged manually in 1:3 ratio once every week.

### Adapting KIND1 cells to feeder-free culture conditions

KIND1 cells were adapted to grow on Geltrex by gradually reducing the HFF density while maintaining recommended concentration of Geltrex. For feeder-free culture, KIND1 cells were grown on 60 mm dish coated with Geltrex as per manufacturer’s instructions in StemPro hESC SFM media supplemented with 10 ng/mL of bFGF (R & D Systems, MN, USA) at 37°C in a humidified atmosphere with 5% CO_2_. Passaging was done mechanically using Cell Lifter (Sigma Aldrich, MO, USA) in 1:3 ratio.

### RT-PCR for evaluating expression of pluripotent markers

Total RNA was extracted using TRIzol reagent (Invitrogen) as per manufacturer’s instructions. DNase I (Invitrogen) treatment was done at 37°C for 30 minutes and spectrophotometric quantification of the extracted RNA was done using Ultrospec 3100 pro (GE Healthcare, PA, USA). The cDNA was synthesized using iScript cDNA Synthesis Kit (Bio-Rad Laboratories, CA, USA) in a 20 μL reaction volume according to the manufacturer’s instructions using GSTORM thermal cycler (Gene Technologies, Braintree, UK). For all the genes, the cycling parameters for PCR comprised of initial denaturation at 94°C for 3 min, followed by 35 cycles of denaturation at 94°C for 30 sec, primer annealing at 62°C for 30 sec and extension at 72°C for 1 min using Dream Taq Polymerase as per manufacturer’s instructions (Thermo Scientific, IL, USA). The primers used for RT-PCR are as described earlier [38]. The PCR products were visualized by electrophoresis on 2% agarose gel, containing 0.5 μg/ml ethidium bromide (Bangalore Genei, Bangalore, India) and the product size was approximated using 100-bp DNA ladder (Bangalore Genei).

### Karyotyping of feeder-free hES cells

KIND1 colonies were treated with 0.2 μg/mL colchicine (Sigma Aldrich) for 3 hrs and harvested using 1× accutase (Invitrogen) followed by treatment with hypotonic solution of 0.075 M KCl (Sigma Aldrich) for 20 min at 37°C. After treatment, cells were pelleted by centrifugation and fixed by addition of cold fixative made of methanol and acetic acid in ratio of 3:1(Qualigens Fine Chemicals, Mumbai, India),followed by G-banding for metaphase analysis. More than 20 metaphases were analyzed to prepare the karyogram.

### Differentiation of KIND1 hES cells

#### Spontaneous differentiation by embryoid body (EB) formation

Embryoid bodies were prepared and cultured as described earlier [38] and EBs were harvested at Day 7 and Day 15. For plating, the EBs (7 days post suspension culture) were plated on gelatin coated dishes for 10 days in same media as used for EBs in suspension culture and later harvested for RNA extraction.

#### Directed differentiation of feeder free KIND1 cells into pancreatic lineage

The protocol published earlier by Kroon et al. [51] was used to differentiate hES cells into pancreatic endoderm with slight modifications. KIND1 feeder free cells showing 80% confluency were cultured using RPMI 1640 media containing 100 ng/mL activin A (R & D Systems), 1 mM sodium butyrate (Sigma Aldrich), and 25 ng/mL wnt-3a (R & D Systems). After 24 hours, 0.2% fetal bovine serum (FBS) was added to RPMI media along with 100 ng/mL activin A, 0.5 mM sodium butyrate. On days 3 & 4 the RPMI media was supplemented with 2% FBS and 100 ng/mL activin A. From day 4 onwards, the basal medium used was DMEM F12 supplemented with 1× B27, 2 μM retinoic acid (Sigma Aldrich), 50 ng/mL noggin (R & D Systems), 0.25 μM cyclopamine (Sigma Aldrich) for 4 days. During the last stage of differentiation, the differentiating cells were cultured in DMEM along with 1× B27 supplement, 2 μM retinoic acid, 50 ng/mL noggin, 1 mM nicotinamide (Sigma Aldrich), 1× non-essential amino acids and 25 μg/mL FGF-10 (R & D Systems).

### qRT-PCR analysis to monitor differentiation of hES cells and PcG expression pattern

RNA extraction for qRT-PCR from differentiated and undifferentiated KIND1 cells followed by cDNA synthesis was done as described in RT-PCR section*.* Quantitative RT-PCR was performed using CFX96 Real Time Machine (Bio-Rad) and iQ SYBRGreen SuperMix (Bio-Rad). The threshold values (Ct) were obtained from CFX 96 manager software (Bio-Rad) and normalized using housekeeping gene 18S Ct values (Comparing 18S Ct values from different time points and passages showed a maximum standard deviation of 2.05 and coefficient of variance < 19%, with no significant difference between the various time points). Efficiency of all the primers used in this study was determined by Standard curve method using 10-fold serially diluted cDNA (mixture of undifferentiated and differentiated KIND1 RNA) and was found to be between 90-110%. The primer sequences are given in Table 1. The amplification conditions comprised of initial denaturation at 95°C for 5 min, followed by 40 cycles of denaturation at 95°C for 10 sec, annealing at 62°C for 20 sec and elongation at 72°C for 30 sec. The fluorescence emitted at each cycle was captured and the melt curve analysis was performed at the end of 40 cycles to determine the homogeneity of the amplified products. The fold change in expression was calculated by 2^-∆∆Ct^ method. Each reaction was carried out in duplicate using samples from 5 biological replicates, 3 technical replicates were performed.

**Table 1:** List of all the primer sequences used for qRT-PCR analysis

**Gene**	**Primer sequence 5′- 3′**	**Size**
18S rRNA	F- GGAGAGGGAGCCTGAGAAAC	171 bp
	R - CCTCCAATGGATCCTCGTTA	
*BMI1*	F - ACGATGCCCAGCAGCAATGACT	172 bp
	R- AAGTGGACCATTCCTTCTCCAGGT	
*CBX2*	F- GAGTACCTGGTCAAGTGGCG	215 bp
	R- TCGGGTTCCTTGAGCTTGGA	
*CERBERUS*	F - GTGCCCTTCAGCCAGACTATAACC	107 bp
	R - TGCGCGGCTCCAGGAAAATG	
*CXCR4*	F - GGCAGCAGGTAGCAAAGTGACGC	334 bp
	R - AGAGGAGGTCGGCCACTGACA	
*E CADHERIN*	F- CACTGGGCTGGACCGAGAGAGTT	236 bp
	R- ACGCTGGGGTATTGGGGGCA	
*EED*	F – TGCCATTGTGTGCTGGAAACCTGG	163 bp
	R - GCCCAATGCAAGCATCTTTTGCC	
*EOMESODERMIN*	F - TTCTGGCTTCCGTGCCCACG	103 bp
	R - CATGCGCCTGCCCTGTTTCGTA	
*EZH2*	F- ACACGGGGATAGAGAATGTGGGTT	455 bp
	R -TCCGCTTATAAGTGTTGGGTGTTGC	
*FOXA2*	F - AGGAGGAAAACGGGAAAGAA	134 bp
	R - CAACAACAGCAATGGAGGAG	
*HAND1*	F- TGCCTGAGAAAGAGAACCAG	273 bp
	R- ATGGCAGGATGAACAAACAC	
*HNF4A*	F - CCCAGCCCCCTAAGAGAGCAC	245 bp
	R - CCCAGCCCCCTAAGAGAGCAC	
*JARID2*	F – TGCACAAGCCGCAGGACTCG	356 bp
	R - ACAGCCCCCTATGAGCGGGAG	
*MAP2*	F- GCATGAGCTCTTGGCAGG	192 bp
	R - CCAATTGAACCCATGTAAAGCC	
*MIXL*	F - AGTCCAGGATCCAGGTATGGTTCC	192 bp
	R- CAACCCCGTTTGGTTCGGGC	
*N CADHERIN*	F - AACTGGGCCAGGAGCTGACCA	122 bp
	R- GTGCCCTCAAATGAAACCGGGCT	
*NANOG*	F - AGTCCCAAAGGCAAACAACCCACTTC	164 bp
	R- TGCTGGAGGCTGAGGTATTTCTGTCTC	
*NKX6.1*	F- CAGAGAGTCAGGTCAAGGTCTGGT	260 bp
	R– CTCGGACGCGTGCAGTAGGA	
*OCT4A*	F - AGCCCTCATTTCACCAGGCC	448 bp
	R- TGGGACTCCTCCGGGTTTTG	
*P19*	F- TTGCAGGCCGCCAGTG	289 bp
	R- GGGTGTCCAGGAATCCAGTG	
*PDX1*	F- GAAAGGCCAGTGGGCAGGCGG	137 bp
	R- GGCGCGGCCGTGAGATGTAC	
*REX1*	F- GGCAAACCCACCCCACTCACC	146 bp
	R- CAAACACCTGCTGGACTGTGAGC	
*RING1A*	F- AGCCCTACGGAGCGGGAACA	258 bp
	R- GGTATCGGCCGCCTCACACG	
*RING1B*	F- CCATGAACAGACTGCAGCGA	125 bp
	R- ACTAGGGCCTGCTTCCTGAT	
*SOX17*	F - AAGGGCGAGTCCCGTATC	221 bp
	R- TTGTAGTTGGGGTGGTCCTG	
*SOX2*	F- GGGGGAAAGTAGTTTGCTGCCTCT	135 bp
	R- TGCCGCCGCCGATGATTGTT	
*SOX9*	F- GACGCTGGGCAAGCTCTGGAGACTT	141 bp
	R- TTCTTCACCGACTTCCTCCGCCG	
*SUZ12*	F- CTGTGGAGGGGGTGGCAGTTAC	144 bp
	R- AGGCCTGGAGGAAAAGCTCGTG	

The expression of gene transcripts specific for definitive endoderm (Day 4), pancreatic gut tube (Day 8), pancreatic progenitor (Days12-16), PRC1 and PRC2 gene transcripts in differentiated cells is expressed relative to undifferentiated KIND1 cells (set as 1).

For expression of PcG gene transcripts in adult human pancreatic RNA (Clonetech Laboratories Inc., CA, USA) and KIND1 cells (cultured on HFF and on feeder free system) the PcG gene transcript, Cts were normalized using housekeeping gene 18S Cts, expression shown is relative to highest detectable (Ct 40). Mean ∆Ct values from qRT-PCR analyses were compared using the unpaired, two-tailed Student’s t-test, p values <0.05 were considered significant, error bars in graphs represent ± Standard Error of Mean. The Co-efficient of variance was found to be ≤ 27%.

### Western blotting to assess pluripotency, differentiation and PcG expression

Undifferentiated and differentiated KIND1 feeder free cells were harvested for protein extraction using ice cold cell lysis buffer containing 50 mM Tris (SRL, Mumbai, India), 1 mM EDTA (Fischer Scientific, NY, USA),150 mM NaCl (Sigma Aldrich), 1 M NaF (Fischer Scientific), 0.1% SDS (Fischer Scientific), 1% triton X-100 (Sigma Aldrich), 2 mM PMSF (Sigma Aldrich) and containing Protease Inhibitor Cocktail (Roche Diagnostics GmbH, Manheim, Germany). The cell lysate was agitated on ice for 30 minutes followed by centrifugation to collect the supernatant. For extraction of histone proteins protocol followed was as described by de Napoles *et al.* 2004 [16]. Protein concentration was estimated by Folin-Lowry method using spectrophotometer (Beckman Coulter Inc, IN, USA). The extracted protein was incubated in Laemmli buffer for 10 minutes at 95°C. 10–20 μg of protein was loaded onto 10% or 15% SDS-PAGE followed by transfer onto PVDF membrane (Amersham Biosciences, Bucks, UK). The blot was blocked with 5% NFDM in 1XTBST overnight at 4°C. Primary antibody mouse anti OCT-4 (1:500, Millipore, CA, USA), rabbit anti SOX17 (1:2000, Millipore), rabbit anti SOX9 (1:500, Millipore), mouse anti PDX1 (1:20000, Sigma Aldrich), rabbit anti EZH2 (1:2500, Epitomics, California, USA), rabbit anti SUZ12 (1:2000, Millipore), rabbit anti BMI1(1:3000, Epitomics), mouse anti H3K27me3 (1:5000, Abcam, MA, USA), Mouse Anti H2AK119ub1 (1:2000, Millipore) were incubated at RT for 2 hours followed by incubation with goat anti mouse/rabbit HRP conjugated secondary antibody (1:5000, Millipore) for 2 hours at RT. Later blot was striped using stripping buffer (62.5 mM Tris, 2% SDS,100 mM β-mercaptoethanol) for 10 minutes at 60°C. Mouse anti β ACTIN (1:5000, Millipore) and rabbit anti-Histone H3 (1:3000, Biolegend, CA, USA) were used as housekeeping protein for normalization. The blots were imaged using Super Signal West Femto substrate (Thermo Scientific) and photographic films (Eastman Kodak Co, USA). Densitometric analysis was done using ImageJ software (http://rsbweb.nih.gov/ij/).

## Author’s contributions

PP involved in study design, carried out experiments, performed data analysis and manuscript preparation. PN involved in data interpretation and manuscript preparation. DB involved obtaining funds, in data interpretation and manuscript preparation. All authors read and approved the final manuscript.
